# Analysis of Genetic Mutations in a Cohort of Hereditary Optic Neuropathy in Shanghai, China

**DOI:** 10.1155/2017/6186052

**Published:** 2017-12-04

**Authors:** Dekang Gan, Mengwei Li, Jihong Wu, Xinghuai Sun, Guohong Tian

**Affiliations:** ^1^Department of Ophthalmology, Eye, Ear, Nose and Throat Hospital of Fudan University, Shanghai, China; ^2^Key Laboratory of Visual Impairment and Restoration of Shanghai, Shanghai, China; ^3^State Key Laboratory of Medical Neurobiology, Institute of Brain Science, Fudan University, Shanghai, China

## Abstract

**Purpose:**

To evaluate the clinical classification and characteristics of hereditary optic neuropathy patients in a single center in China.

**Method:**

Retrospective case study. Patients diagnosed with hereditary optic neuropathy between January 2014 and December 2015 in the neuro-ophthalmology division in Shanghai Eye and ENT Hospital of Fudan University were recruited. Clinical features as well as visual field, brain/orbital MRI, and spectrum domain optical coherence tomography (SD-OCT) were analyzed.

**Results:**

Eighty-two patients diagnosed by gene test were evaluated, including 66 males and 16 females. The mean age of the patients was 19.4 years (range, 5–46 years). A total of 158 eyes were analyzed, including 6 unilateral, 61 bilateral, and 15 sequential. The median duration of the disease was 0.5 year (range, 0.1–20 years). Genetic test identified 68 patients with Leber hereditary optic neuropathy, 9 with dominant optic neuropathy, and 2 with a Wolfram gene mutation. There was also one case of hereditary spastic paraplegia, spinocerebellar ataxia, and polymicrogyria with optic nerve atrophy, respectively.

**Conclusion:**

Leber hereditary optic neuropathy is the most common detected type of hereditary optic neuropathy in Shanghai, China. The detection of other autosomal mutations in hereditary optic neuropathy is limited by the currently available technique.

## 1. Introduction

Hereditary optic neuropathy (HON) is one of the major eye diseases leading to visual impairment in children and adolescents. Due to differences in patient age, gender, and genetic background, there are variations in the HON classification system. It is well established that autosomal dominant hereditary optic neuropathy (ADOA) is the most common type of HON, followed by Leber hereditary optic neuropathy (LHON) and other plus syndromes associated with damage of the nervous system [1, 2]. This study analyzed 82 patients diagnosed with HON by routine screening and a blood genetic test to provide new insights on the diagnosis and gene mutation spectrum in China.

## 2. Materials and Methods

### 2.1. Samples

Patients with decreased vision and optic atrophy were recruited from Shanghai Eye and ENT Hospital of Fudan University, division of neuro-ophthalmology, between January 2014 and December 2015. The clinical data were retrospectively analyzed. This study was approved by the Clinical Research Ethics Committee of the hospital, and all patients enrolled gave written formal consent.

The patient inclusion criteria were as follows: (i) acute, chronic, or occult vision loss; (ii) ophthalmic examinations showed color vision and/or visual field defect, if unilateral optic neuropathy with relative afferent pupillary defect (RAPD); (iii) head/orbital MRI for excluding compressive disorder; (iv) blood tests negative for hepatitis B, syphilis, tuberculosis, and other infectious diseases; and (v) a clear association between optic neuropathy and gene mutations.

The patient exclusion criteria were as follows: (i) traumatic optic atrophy; (ii) optic neuritis or steroid treatment improving visual acuity dramatically; and (iii) incomplete clinical data or patients refusing to sign the consent form.

### 2.2. Research Approach

Demographic characteristics, family hereditary disorder history, duration of visual loss, and whether affected eyes were unilateral or bilateral were recorded. Clinical examinations of visual function are as follows: best-corrected visual acuity (BCVA), fundus optic disc morphology, static Humphrey or Octopus visual field, and dynamic Goldmann perimetry. Hematological tests are as follows: erythrocyte sedimentation rate (ESR), c-reactive protein (CRP), rheumatoid factor (RF), antistreptolysin (ASO), antinuclear antibodies (ANA), extractable nuclear antigen (ENA), antineutrophil neutrophil cytoplasmic antibody (ANCA), toluidine red syphilis serum test (TRUST), treponema pallidum specific antibodies (TPPA), and aquaporin 4 antibody (AQP4-IgG) were performed.

### 2.3. Genetic Test

3–5 *μ*g DNA were extracted from blood following the second-generation sequencing capture protocol and amplified to establish the genomic library. The target genes were screened out and sequenced by Illumina HiSeq 2000 sequencing system. The average depth was no less than 200x. In the following analysis, we focused on mitochondrial gene mutation hot spots (59 sites), dominant optic atrophy gene (OPA1–8), external ophthalmoplegia and Wolfram (WFS) hotspot mutations, retinitis pigmentosa, macular degeneration-related genes, and rare sites including morning glory, small eye, and open-angle glaucoma.

### 2.4. Statistics

All data were analyzed by SPSS11.5 software. The normal distribution of the data was tested and presented as the mean ± standard deviation (X ± S) or median. Data counts were presented as a percentage.

## 3. Results

### 3.1. Demographic and Clinical Characteristics

Of 117 patients suspected with HON, 82 cases (66 males and 16 females) were obtained after the exclusion of cone dystrophy, occult macular degeneration, Leber congenital amaurosis, congenital stationary night blindness, glaucoma, and other common causes of child and adolescent neuropathy, retinopathy, or maculopathy. The mean age of the patients was 19.4 years (range, 5–46 years old). One hundred fifty-eight effected eyes were evaluated, including 6 patients affected with unilateral, 61 bilateral, and 15 sequential neuropathies. The median duration of the visual decrease was 0.5 year (range, 0.1–20 years). The initial visual acuity after the attack was from hand motion to 0.8, while the mean prognosis visual acuity is 0.1.

### 3.2. Visual Field Damage

Of 136 eyes from 68 patients that underwent visual field examination, central scotoma accounted for 69.9%, cecocentral scotoma connected with blind spot for 8.0%, paracentral and temporal defect for 6.6%, and diffuse defect for 15.5%. Interestingly, 81.5% of the asymptomatic eyes also demonstrated the central visual field damage.

### 3.3. Gene Mutation Types

**Figure 1:**
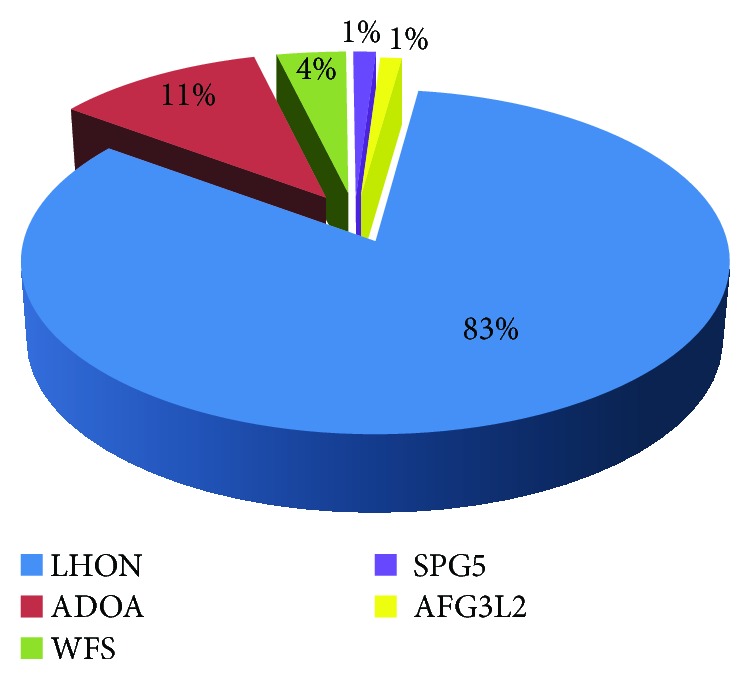
The genetic testing profile of patients with hereditary optic neuropathy.

Of the 82 cases, 68 were identified with LHON, accounting for 83% of the cases. There were nine cases with ADOA, 8 cases with OPA1 mutation, and one case with OPA3 mutation. As for the other rare mutations, there were three cases with Wolfram (WFS mutations): two cases with type 1 (WFS1) gene mutation (p.G576S) and one case with type 2 (CISD2) gene mutation (p.L91fs). One case with hereditary spastic paraplegia (SPG5) gene mutation and one case with spinocerebellar ataxia type 28 (AFG3L2) gene mutation (p.R632X). Both of them were with optic atrophy. There was also one case of polymicrogyria with optic nerve hypoplasia (TUBA8 gene mutation, p.Q235R).  Figure 1 shows the disease spectrum.

**Figure 2:**
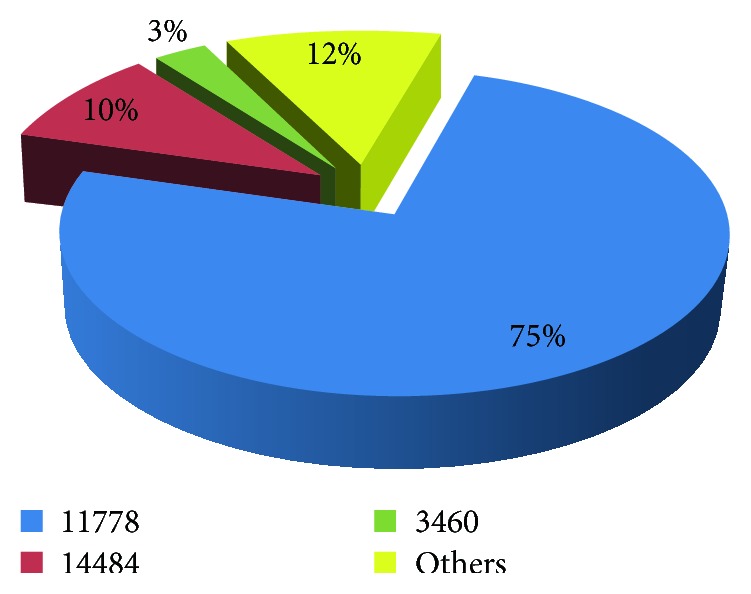
The mitochondrial DNA mutation profile of patients with LHON.

For the 68 patients with LHON, mitochondrial mutations identified that the primary mutations sites of 11778, 14484, and 3460 accounted for 75%, 10%, and 3%, respectively. Other rare gene mutation sites at 11696, 8344, 3700, and 3635 account for the other 12% of mutations (Figure 2).

## 4. Discussion

**Figure 3:**
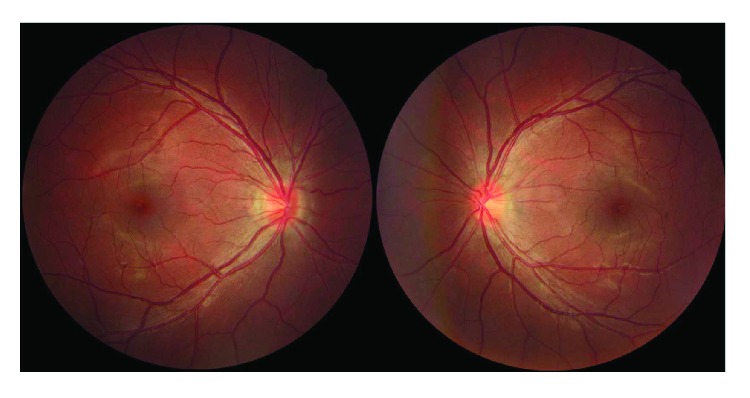
A 30-year-old male with bilateral decreased vision after taking ethambutol for treatment of tuberculosis for 6 months. The fundus shows optic disc hyperemia and he was identified as having mtDNA 11696 mutation.

**Figure 4:**
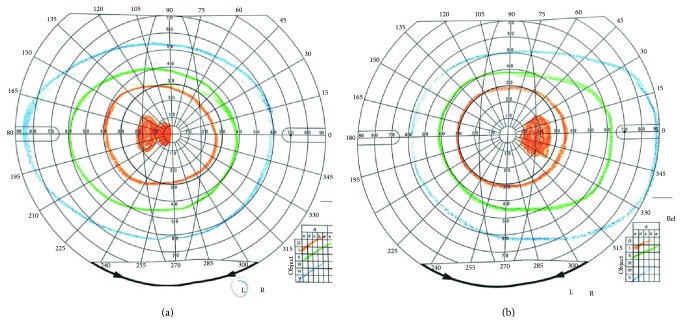
The Goldmann perimetry of the patient from Figure 3 showing bilateral cecocentral scotoma, which is difficult to distinguish from the toxic metabolic optic neuropathy.

HON is the most common cause of vision loss and optic nerve atrophy in children and adolescents. Wei et al. demonstrated that HON was more prevalent in children younger than 14 years of age [3, 4]. We also described the clinical features of LHON patients in an earlier study [5]. In this study, we found that LHON accounts for the largest proportion of the cases presented to ophthalmologists and the three primary mutations (11778, 14484, and 3460) of mitochondrial are the major mutation type, although the 3460 mutation showed a considerable low incidence in Shanghai, China. Other rare mutations occurred at 11696, 3635, 3800, 8344, and 14692. After evaluating the clinical manifestation of some rare gene mutation patients, we found that the 11696 (A-G) mutation is associated with older individuals who drink or take toxic medications and decreased vision was from very mild to devastating. Although the 11696 mutation was reported, which encodes ND4 protein that is similar with 11778, the clinical manifestations of our patients are variable [6, 7]. In particular, one patient had decreased vision after taking ethambutol for antituberculosis therapy (Figures 3 and 4) and another comorbidity with neuromyelitis optica.

There is one case of mtDNA8344 mutation. This patient was diagnosed with optic nerve atrophy but without myoclonic seizures and any other encephalopathy association. These clinical characteristics do not match MERRF syndrome, which results from this mutation site [8]; we speculate that this patient was inflicted with an early or mild type of the disease.

**Figure 5:**
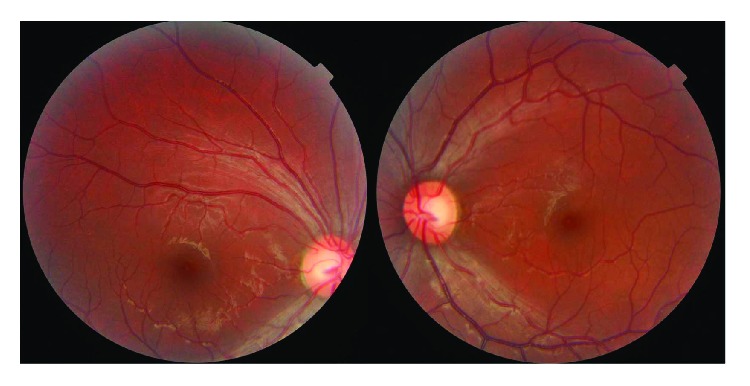
A 24-year-old male with bilateral decreased vision and detected mtDNA 11484 mutation. The fundus shows a pale bilateral disc and the thinning of the retinal nerve fiber layer.

**Figure 6:**
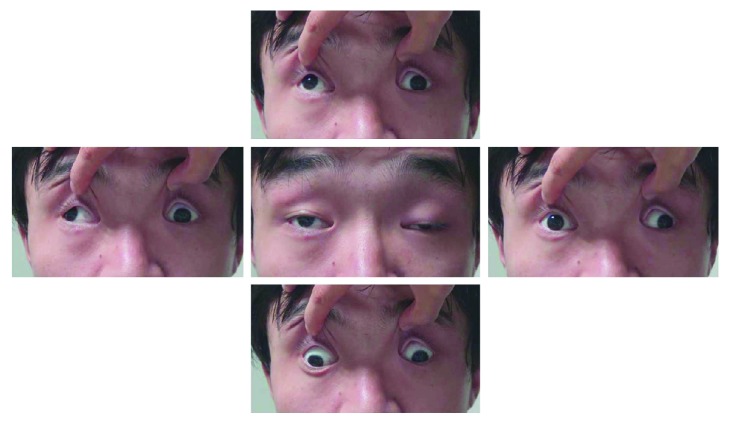
The patient from Figure 5 showing the ptosis and extraocular muscle paralysis.

In another case of the mtDNA14484 mutation, the patient exhibited external ophthalmoplegia combined with bilateral decreased visual. However, other genes associating with ophthalmoplegia were not detected (Figures 5 and 6). In another patient with 14484 mutation, the central serous retinopathy was the presenting symptom followed by decreased visual acuity and optic nerve atrophy.

There was one patient with the 11778 mutation which showed mental retardation and leukoencephalopathy. We considered it as Leber plus syndrome [9, 10].

It was reported that dominant HON accounts for the largest proportion [2]. However, Chen et al. [5] reported that the OPA1 had a lower mutation rate, compared to mitochondrial mutations in optic neuropathy. In this study, we detected nine ADOA mutations, with eight OPA1 mutations and one OPA3 mutation. With regard to the discrepancy, we believe that the following factors are related: (1) the onset vision decrease in children, especially unilateral, could not be reported immediately and was only found during school age; (2) insidious onset compared with LHON, without the acute attack; (3) temporal disc pallor is often diagnosed and treated as glaucoma; and (4) compared to mitochondrial mutations, autosomal genetic test is more complex. Some patients with OPA mutations also exhibit peripheral neuropathy, deafness, and other neurological abnormalities, which is consistent with the ADOA plus syndrome.

One rare case with Wolfram syndrome type II mutation presented with bilateral optic nerve atrophy. His elder brother died at 17 years of age due to ketoacidosis. The further examination revealed diabetes insipidus, diabetes mellitus, and deafness. In addition, there is one case of optic nerve hypoplasia combined with polymicrogyria, which is consistent with the literature [11].

In conclusion, mitochondrial DNA mutations are most commonly found in patients with HON in Shanghai, China. The detection of ADOA is limited by the currently available diagnostic tests in China. The LHON patients usually presented with decreased vision, other peripheral neuropathy syndromes, and muscle and central nerve system disorders. The optic atrophy can also be taken as one of the central nerve system defects.
